# Does a lower self-concept contribute to mental health disparities of diverse immigrant youth from middle childhood to late adolescence?

**DOI:** 10.1186/s40359-021-00555-0

**Published:** 2021-04-23

**Authors:** J. Busch, C. Claus, S. Schneider, R. G. Siefen

**Affiliations:** 1 Faculty of Psychology, Department of Child and Family Research, Ruhr-University, Bochum, Germany; 2 Faculty of Psychology, Department of Clinical Child and Adolescent Psychology, Ruhr-University, Bochum, Germany; 3 University Children’s Hospital, Ruhr-University, Bochum, Germany

**Keywords:** Self-concept, Depression, Anxiety, Immigrant, School, Germany

## Abstract

**Background:**

Three out of ten children in Germany have immigrant backgrounds and this proportion is expected to further increase in subsequent years. While immigrant youth have been found more vulnerable to developing symptoms of depression and anxiety, the underlying mechanisms of how such disparities unfold during youth development are still understudied. Some previous research has found that immigrant youth are at risk of experiencing a less positive self-concept compared to non-immigrant youth. We investigated whether the self-concept mediates mental health disparities and explored variability in such associations from middle childhood to late adolescence.

**Methods:**

Overall 1839 children and adolescents aged 6–21 years (*M* = 14.05 years, *SD* = 3.03, 49.8% female, *n* = 782 with immigrant status) participated in a cross-sectional self-report survey in classroom settings using scales from the Beck Youth Inventories II (Beck et al. in Beck Youth Inventories - Second Edition, Psychological Corporation, San Antonio, 2005) to assess self-concept and symptoms of depression and anxiety. Links between immigrant status, age, self-concept and symptom levels of depression as well as anxiety were examined using hierarchical regression and moderated mediation models.

**Results:**

Immigrant youth reported higher symptom levels of depression and anxiety than their non-immigrant peers but did not differ in their self-concepts. Hypothesized moderated mediation models were not fully supported and self-concept neither mediated the link between immigrant status and depression nor immigrant status and anxiety. However, self-concept was a significant predictor for symptom levels of depression as well as anxiety, with stronger associations in adolescents.

**Conclusions:**

Our study substantiates previous findings that immigrant youth in Germany have overall increased symptom levels of depression and anxiety compared to non-immigrant youth. Our study however does not support that immigrant youth have a more negative self-concept and that the self-concept mediates such internalizing mental health disparities. Findings match previous evidence that developing a positive attitude towards the self is linked to better mental health. Beyond that, our findings suggest that mental health interventions addressing the self-concept could be especially relevant when targeting adolescents. Further research is needed to deepen the understanding of the mediating processes between migration status and mental health variables.

## Background

About one-fifth of all children and adolescents in Germany experience mental health problems [2, 3] with symptoms of depression and anxiety being among the most common in these age groups [4]. Evidence is alarming, as developing a clinically relevant anxiety or depressive disorder during childhood and adolescence impairs the unfolding of developmental potentials and increases the risk for mental health problems during adulthood [2]. Notably, a higher risk for developing such common internalizing mental health problems has been reported for diverse groups of children and adolescents from immigrant families residing in European countries [5, 6] including newly arrived refugees [7] and those, whose families have lived in the resettlement country for the second or even third generation [8]. Symptoms associated with depression and anxiety have been consistently linked to severe psychological and social consequences among immigrant youth; Plener et al. [9] found a higher prevalence of suicide attempts compared to their non-immigrant counterparts, van Oort et al. [10] substantiated links to the emergence of socio-economic disparities between immigrant and non-immigrant populations during adulthood.

With a growing number of children and adolescents in Germany, currently 36%, have an immigrant status [11], research on the foundations of systematic differences in the mental health status between immigrant and non-immigrant youth is becoming increasingly relevant. Specifically, a better understanding of distinctive mechanisms is an important step to mitigate mental health disparities within the diverse societies of tomorrow. A literature review including twenty global studies on immigrant youth in resettlement countries [12] suggested a lower socio-economic status as a mediator for increased levels of mental health problems among young immigrant populations. However, the reviewed studies also supported that the relations between holding an immigrant background and experiencing mental health problems often persisted above and beyond the explanatory effects of socio-economic status [12]. Some of the reviewed evidence demonstrated within-family stress between first- and second-generation immigrants to additionally account for mental health problems in immigrant youth. Comparing the reviewed studies on within-family conflicts between first- and second-generation immigrants yielded that such findings could be at least partially confounded with age [13]. When immigrant youth are compared to non-immigrant youth of the same age group disparities seem to diminish [14]. Within-family stress is thus more likely related to a specific developmental period and the specific effects linked to immigrant background still remain to be delineated further. Previous findings on the mediating processes were moreover critically discussed as studies were conducted in different and also heterogenous populations, applied varying methodology and, even partially, yielded inconsistent evidence. For example, both lower and higher levels of mental health problems in immigrant youth when compared to non-immigrant youth were found in some studies [15, 16]. One methodological critique addressed the absence of univocal definitions of key constructs such as “immigrant background” or the measurements for mental health problems in immigrant youth [12].

Another recent literature review distinctively studied potential mechanisms and discussed pathways of how mental health disparities between immigrant and non-immigrant populations could unfold [17]. Determinants such as language proficiency, sex, age and, again, socio-economic status were found to be linked to differences in health literacy among immigrant and non-immigrant populations that subsequently are linked to mental health disparities. Authors of that review additionally discussed how heightened access barriers within healthcare systems disadvantage immigrant populations (i.e., less health care benefits/ less opportunities to develop health literacy) and thus likely contribute to mental health disparities [17]. Other studies adopting ecocultural perspectives found cultural distance to the host community [18, 19], social stress [6] and acculturative stress [20] to be linked to increased levels of especially internalizing mental health problems among immigrant youth.

Previous studies on the determinants and mechanisms of mental health disparities in immigrant youth so far have less focused on the question of how those disparities unfold within-person and have seldom considered differences across developmental periods. To shed light on such genuine developmental psychopathological processes, this study investigates the self-concept as a correlate for internalizing mental health problems during youth development. Self-concept is defined as the overall system of beliefs, cognitive and affective attitudes towards oneself. The term summarizes a person’s self-evaluation regarding specific aspects such as one’s academic abilities, physical performance or self-regulation capacities [21, 22]. The self-concept constitutes intrinsic cognitive schemata mainly determined by feedback from social environments [23]. Self-directed feedback is processed intra-personally in order to form a coherent picture of oneself. Previous research [24] has described two strategies of how the self-concept can be structurally organized, namely *compartmentalization* and *integration. Compartmentalization* describes a self-image that is structured in different categories containing either positive or negative components of oneself, while *integration* forms a self-concept that consists of positive as well as negative information about the self [24]. Evidence that links the structure of the self-concept to self-esteem suggests that individuals who tend to compartmentalize their self-concept may experience difficulties in accessing coherent information about oneself, leading to an overall more negative evaluation of the self [25].

A negative and unstable self-concept has also been linked to symptoms of anxiety and depression among children and adolescents in general populations [26–30]. For example, a self-report survey with children aged 9 to 13 years in school settings showed that those with symptoms of depression and anxiety also reported lower levels of global self-esteem and a lower quality of life. Such links were stronger in older children of the sample [31]. However, no study has yet focused on the self-concept’s distinctive significance for internalizing mental health problems among immigrant youth. Some previous evidence supports the notion that immigrant children and adolescents are more vulnerable to a lower self-concept [32]— although such evidence is still limited and remains inconsistent. Conceptually, immigrant status is a critical biographical characteristic for children and adolescents as it is linked to intra- and interindividual processes of adaptation and to an increased risk of experiencing stigmatization [33]. Even for those immigrant children and adolescents without personal migration experiences, their origin culture as conveyed by relatives and the migration experience of parents or grandparents still forms the children’s and adolescents’ self-ascriptions [33, 34]. Socio-cultural discontinuities that immigrant youth likely experience between the family context and the host country’s society can impede the formation of a positive and integrative self-concept throughout youth development [35, 38, 39]. While immigrant children and adolescents are exposed to the host countries’ influences in school contexts and peer-networks, they are often taught the language, traditions and values of their origin country at home [36]. Such potentially opposing contexts can result in the development of a fragmented "self-culture" whose principles are ambiguous, context-dependent, conflicting and which can be accompanied by symptoms of withdrawal and self-insecurity [8, 37]. The fragmentation could thus promote a compartmentalized structure of the self-concept.

Adding a developmental perspective, an inner focus on the emerging self-concept arises with the beginning of puberty and peaks during adolescence [38]. Adolescence marks a hypersensitive period to all kinds of social stimuli when a coherent self-concept is formed via information seeking [40, 41]. During that period, schools provide a central social context to establish a coherent self-concept. Since immigrant youth are at heightened risk to experience cultural discontinuities between school and non-school contexts, forming a coherent and positive self-concept in schools could be particularly challenging for this group [42, 43]. Reviewing the research on immigrant children and adolescents from different generations in school-contexts, previous work has mainly focused on the academic components of the self-concept. For example, Mitchell [44] overall found a lower academic self-concept for immigrant adolescents with Central American backgrounds attending schools in the United States and substantiated links to lower levels of well-being. The author, however, did not find differences regarding the generational status of immigration (i.e., first-, second- and third-generation). In further studies a lower self-concept was correspondingly considered a socially-determined and multi-directional pathogenic influence [45, 46], also impacting internalizing mental health problems [47]. It is therefore necessary to extend research on the self-concept among immigrant youth to self-directed cognitions and emotions within school settings.

**Fig. 1:**
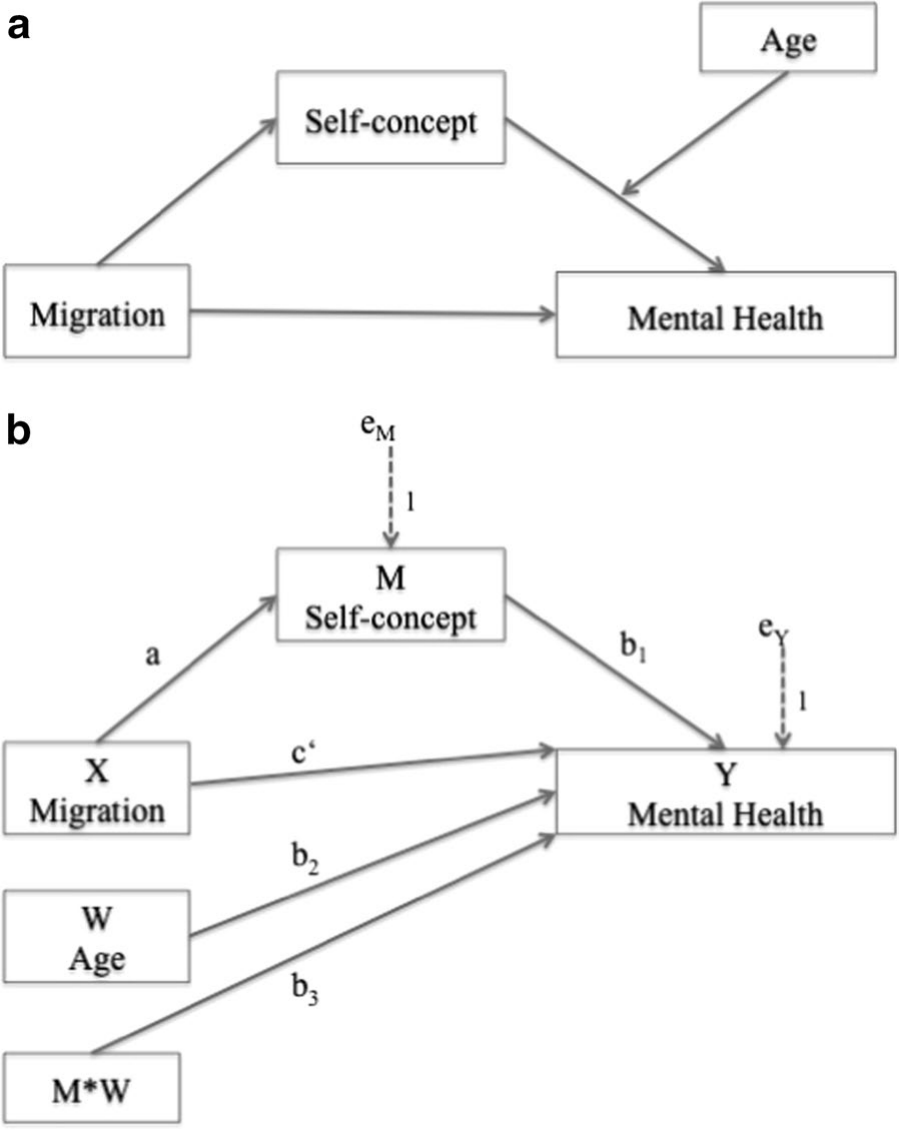
Panel **a** illustrates the moderated mediation model according to our study hypotheses. We proposed that immigrant youth report increased symptom levels of depression and anxiety (H1), lower self-concept predicts higher symptom levels (H2), self-concept mediates the link between immigrant status and symptom levels (H3) and that age moderates the hypothesized mediation (H4). Panel **b** illustrates the statistical realization as suggested by Hayes [56] and corresponds to the analyses reported in Table 6

Investigating the role of the self-concept throughout youth development, our study goal was to better understand the foundations of symptoms of depression and anxiety in immigrant children and adolescents. Specifically, our study hypotheses were to replicate previous findings that (H1) immigrant children and adolescents experience higher symptom levels of depression and anxiety than non-immigrants in school settings, and (H2) that a lower self-concept was linked to those increased symptom levels. Based on this replication, we expected (H3) the self-concept to mediate the respective links between immigrant status and depression as well as anxiety, and (H4) that the effects on symptom levels are stronger during adolescence. The theoretical model reflecting the study hypotheses is illustrated in Fig. 1a.

## Methods

### Study design and sample

Overall, *N* = 1907 children and adolescents participated in a cross-sectional self-report survey that we conducted in primary and secondary schools across Germany’s most populous and multicultural federal state, North-Rhine Westphalia. We chose schools in different regions (i.e., varying the degree of urbanization) and neighbourhoods and considered all types of German secondary schools to balance the student sample. At study enrollment, teachers informed parents using written handouts in several languages. Parents who agreed to letting their children participate in the study signed an informed consent. All students in primary and secondary school classrooms were eligible to participate if they were regular members of the class, had a minimum of second grade reading comprehension and presented their parental declaration of consent. We intended to classify our full study sample post-hoc into a non-immigrant and an immigrant group based on their own or familial history of migration. Data were collected via classroom surveys according to a standardized administration protocol. No more than 8 participants per study facilitator were assessed at once in secondary schools. For primary school children, group surveys with a maximum of 4 children per study facilitator were conducted to increase levels of assistance, when necessary. Due to difficulties in the recruitment of primary schools, additional data collections were conducted in sports and social clubs for younger children following the same procedure. All study facilitators were advanced undergraduate or graduate students in psychology, supervised by clinically experienced researchers.

### Measures

The study questionnaire comprised a demographic section including participants’ age, sex, migration status and the indicators for parents’ socio-economic status. The highest number of parental school years and parents’ profession according to the International Socio-Economic Index of Occupational Status [48] were used to assess the familial socio-economic status of the sample. To measure participants’ internalizing mental health problems, subscales of the German version of the Beck Youth Inventories-II (BYI-II) were used [49]. The BYI-II is a set of overall 5 self-report questionnaires with 20 items each for assessing depression, anxiety, self-concept, anger and disruptive behaviour in children and adolescents aged 7–18 years. Depending on children’s age and reading competence, each questionnaire takes about 8–10 min for completion. Participants responded on a 4-point Likert scale (0, “never” to 3, “always”) to rate how frequently each statement applied to their daily cognitions, emotions and behaviours during the past two weeks. The BYI-II is designed for a large age range, as it operationalizes symptoms in easy language. That also facilitates the use of the BYI-II for immigrant groups, whose German language skills are often on average lower. In accordance with our study aims, the BYI-II questionnaires on depression, anxiety and self-concept were used in this study. We rigorously adhered to the BYI-II manual for scoring and missing value handling.

#### Self-concept

The positively worded self-concept inventory (Beck Self-Concept Inventory for Youth, BSCI-Y) assesses cognitive and emotional perceptions of competency and self-worth. It comprises the overall system of self-directed perceptions, cognitive and affective attitudes toward one's own person such as the perceived social role (e.g., "People want to be with me"), the personal strengths (e.g., "I am good at telling jokes") and self-acceptance (e.g., " I like myself "). The inventory showed good internal consistency (Cronbach’s *α* for immigrants = 0.86; Cronbach’s *α* for non-immigrants = 0.86).

#### Depression

The depression inventory (Beck Depression Inventory for Youth, BDI-Y) screens for symptoms of depression in line with the established criteria of the Diagnostic and Statistical Manual of Mental Health Disorders, Fourth Edition [50]. This included low mood (e.g., "I feel sad"), somatic complaints (e.g., "I have trouble sleeping”) and latent suicidal tendencies (e.g., "I wish I were dead"). The inventory showed excellent internal consistency (Cronbach’s *α* for immigrants = 0.93; Cronbach’s *α* for non-immigrants = 0.93).

#### Anxiety

The anxiety inventory (Beck Anxiety Inventory for Youth, BAI-Y) screens for core symptoms of anxiety disorders relevant to children and adolescents such as general anxiety disorder, panic disorder and social phobia. In line with the Diagnostic and Statistical Manual of Mental Health Disorders, Fourth Edition [49], items reflect participants’ specific fears including in different situations (e.g., “I worry when I am at school”), worried social cognitions (e.g., “I worry people might get mad at me”) and physiological symptoms (e.g., “I get shaky”). The inventory showed very good internal consistency (Cronbach’s *α* for immigrants = 0.89; Cronbach’s *α* for non-immigrants = 0.91).

### Data analyses

We performed data preparations and moderation models using SPSS version 25 [51] and regression models using R Version 3.5.0 [52]. Alpha-error probability was *p* < 0.05 (two-tailed) in all analyses. We adjusted for multiple comparisons using the “Multiple Comparisons Calculator” [53] in accordance with the procedure established by Bonferroni and Holm [54]. All *p*-values, considered in hypothesis testing are displayed in bold in the study tables.  We indicate significance only for those *p*-values after adjustment but consistently report unadjusted *p*-values throughout all tables for coherence in the illustration. For hypotheses on mediation, we interpret bootstrap confidence intervals (CIs). In preliminary analyses, we inspected data and checked statistical assumptions for parametric statistical procedures and computed a correlation matrix on variables considered in main analyses.

#### Hypotheses 1 and 2

For H1, we compared levels of depression, anxiety and self-concept between immigrant and non-immigrant youth while controlling for the covariates age, sex and parental socio-economic status. We therefore calculated two-step hierarchical regression models and, additionally, calculated *R*^*2*^ to determine the effect sizes. For H2, we similarly examined the impact of the self-concept on depression and anxiety, also controlling for age, sex and parental socio-economic status.

#### Hypotheses 3 and 4

We used second stage moderated mediation models [55] to test H3 and H4 as theoretically illustrated in Fig. 1a. We performed modeling using PROCESS, third version, a macro for SPSS developed by Hayes [56] that follows a regression-based approach to integrate mediation and moderation in a conditional process analysis [56]. As displayed in Fig. 1b, and contrary to the widely used historical method by Baron and Kenny [57], the regression-based approach quantifies the indirect effect between the antecedent *X*, the mediator *M* and the consequent *Y* itself and does not require an association between *X* and *Y* as a precondition [56]. The second stage moderated mediation model uses bootstrapping (*k* = 5000) to obtain estimates for 95% bias-corrected CIs for the conditional indirect effects in moderated mediation. The indirect effect is calculated for different conditional values of the moderator variable (i.e., two age groups—youth below or above 14 years of age). Conditional indirect effects are statistically significant when zero is not included between the lower and upper bound of the 95% bias-corrected bootstrap confidence intervals generated for different values of the moderator variable *W*. The bootstrapping method is also superior to the Sobel test [58] due to higher statistical power and independency of the normal distribution of the underlying data [56].

## Results

### Descriptive analysis of the study sample

Of the *N* = 1907 children and adolescents, two did not state their age, another 66 did not complete the demographic section on the immigrant status of their parents or grandparents and were therefore excluded a-priori. This resulted in an effective study sample of *N* = 1839. About half of the participants were female (*n* = 49.8%) and participants were on average 14.05 years old (*SD* = 3.03). A large proportion of the sample comprised immigrant children and adolescents (*n* = 782, 42.5%) with most of these being in the second immigrant generation. Immigrant youth themselves or their families originated from 69 different countries with Turkey (*n* = 194, 10.5%), Poland (*n* = 111, 6.0%), and Russia (*n* = 42, 2.3%) being the most frequently stated countries. Immigrant youth overall came from families with a lower socio-economic status compared to non-immigrant youth. Given the population-based data collection procedure and demographic characteristics of the obtained study population, the immigrant group in our sample reflects the general population with immigrant backgrounds in Germany [47]. The measurements used in this study were created for an age range of 7–18 years, reflecting the schooling period in Germany. Seventy-six participants were outside of the defined age range, that is, 8 participants younger than 7 years and 68 participants older than 18 years. Since these students were members of the classrooms and their exclusion did not yield different results, they remained part of the presented analyses. Note that our effective study sample includes missing values of less than 0.9 percent per analyzed symptom variable. Due to the overall large sample size, we decided to conduct analyses based on that data. Table 1 reports basic socio-demographic information of the effective study sample. Table 2 gives an overview on the symptoms of depression and anxiety and the self-concept of the effective study sample. Table 3 shows correlations between study measures and socio-demographic information.

**Table 1:** Socio-demographic characteristics of the sample

Variable	Immigrant (n = 782)	Non-immigrant (n = 1057)
Sex (female, %)	47.4	51.5
Age in years, mean (SD)	14.08 (2.99)	14.03 (3.06)
Parental SES (%)		
Highest (14–19 years)	23.5	30.4
High (12–13 years)	23.1	30.4
Medium (10–11 years)	35.5	34.1
Low (up to 9 years)	17.9	5.1
ISCED School Type (%)		
ISCED-1 (Primary School)	44.0	32.6
ISCED-2 (Lower Secondary)	31.8	19.1
ISCED-3 (Upper Secondary)	24.2	48.3
Migration status, n (%)		
First Generation	130 (16.6)	–
Second Generation	614 (78.5)	–
Third Generation	38 (4.9)	–

SES, Parental socio-economic status was defined by parents’ highest number of schooling years. The migration status refers to the child or adolescent reporting a history of immigration. For ISCED School Type, we classified schools that youth of our sample attended according to the “International Standard Classification of Education” [74]

**Table 2:** Descriptive analysis of the Beck Youth Inventories (second edition) for symptoms of depression and anxiety by migration status and age

Study group per measure	M	Min	Max	SD	Med	Clin
Depression						
Immigrants						
Children	10.13	0	60	10.56	7	11.51
Adolescents	9.60	0	52	8.90	8	8.95
Non-Immigrants						
Children	8.30	0	56	8.51	6	8.80
Adolescents	8.75	0	47	8.97	6	7.57
Anxiety						
Immigrants						
Children	15.99	0	60	10.51	15	6.27
Adolescents	14.90	0	41	8.51	14	8.70
Non-Immigrants						
Children	12.81	0	60	9.00	12	8.99
Adolescents	13.42	0	56	8.97	11	6.15
Self-Concept						
Immigrants						
Children	38.86	4	60	9.45	40	5.88
Adolescents	38.38	10	59	7.68	39	7.93
Non-Immigrants						
Children	39.47	12	60	9.09	40	8.80
Adolescents	37.89	9	60	7.63	38	6.43

*M*, mean scores. *SD*, standard deviation. *Med*, Median. Four-point Likert scale from *0* (“never”) to *4* (“always”). *Clin*, Participants above clinical cut-off (T-Score of 60 and above) in percent. Children are between 6.92 and 13.92 years, adolescents are between 14.05 and 21.17 years of age. The full sample consisted of *n* = 357 children and *n* = 425 adolescents with immigrant backgrounds, and *n* = 445 children and *n* = 612 adolescents without immigrant backgrounds

**Table 3:** Correlation matrix of depression, anxiety, self-concept, age, sex, SES and migration status

Variable	Depression	Anxiety	Self-concept	Age	Sex	SES	Migration
Depression	1						
Anxiety	0.697	1					
Self-Concept	− 0.456	− 0.283	1				
Age	0.022	− 0.003	− 0.144	1			
Sex	− 0.189	− 0.196	0.128	0.025	1		
SES	− 0.082	− 0.100	0.112	− 0.074	0.095	1	
Migration	− 0.069	− 0.116	0.000	− 0.008	− 0.04	0.217	1

SES, parental highest socio-economic status. Dichotomous variables (Sex, Migration) compared to metric variables using point-biserial correlation. Categorical Variable (SES, 4 groups) compared to metric variables using biserial correlation. Dichotomous and categorical variables compared to each other using Cramer’s *V*

### Examination of Hypotheses 1 and 2

In analyses for H1, the covariates sex and parental socio-economic status predicted symptom levels of depression and anxiety while in regressing self-concept the covariate age likely had additional predictive value. As expected in H1, immigrant children and adolescents reported increased symptom levels of depression (*B*_*Unstandardized*_ = 1.223, adjusted *p* < 0.05) and anxiety (*B*_*Unstandardized*_ = 2.179, adjusted *p* < 0.01) but, contrary to our expectations, did not report a lower self-concept compared to non-immigrants (*B*_*Unstandardized*_ = 0.195, adjusted *p* > 0.05). Beyond the covariates, the migration status explained small additional proportions of unique variance in symptoms of anxiety (*ΔR*^*2*^ = 0.013) and depression (*ΔR*^*2*^ = 0.004; see Table 4). In analysis for H2, we found the self-concept to influence symptoms of depression such that a lower self-concept was linked to increased symptom levels (*B*_*Unstandardized*_ = − 0.477, adjusted *p* < 0.001). Consistently, self-concept also predicted increased levels of anxiety in the same direction (*B*_*Unstandardized*_ = − 0.283, adjusted *p* < 0.001). While the influence of the self-concept on both outcomes was substantial, it was larger on symptom levels of depression (*ΔR*^*2*^ = 0.183) than of anxiety (*ΔR*^*2*^ = 0.063). Details of the stepwise hierarchical regression analyses on H2 are presented in Table 5.

**Table 4:** Hierarchical regression analysis for migration status predicting symptoms of depression, anxiety and self-concept

Variable	Model 1				Model 2			
	*B*	*SE*	*t*	*p*	*B*	*SE*	*t*	*p*
*Depression*								
Intercept	27.732	1.236	22.440	< .001	26.815	1.277	21.003	< .001
Age	0.066	0.070	0.932	.352	0.067	0.070	0.957	.339
Sex	3.624	0.426	8.503	< .001	3.666	0.426	8.612	< .001
SES-medium	− 0.936	0.750	− 1.247	.213	− 0.593	0.759	− 0.781	.435
SES-high	− 1.983	0.772	− 2.569	.010	− 1.548	0.786	− 1.969	.049
SES-highest	− 2.432	0.772	− 3.149	.002	− 2.003	0.786	− 2.549	.011
Migration					1.223	0.439	2.787	.025*
*ΔR*^*2*^	*R*^*2*^ = .046, *F*(5, 1800) = 17.27, *p* < .001				*ΔR*^*2*^ = .004, *F*(1, 1799) = 7.765, *p* = .005			
*Anxiety*								
Intercept	33.395	1.246	26.805	< .001	31.761	1.281	24.792	< .001
Age	− 0.010	0.071	− 0.139	.890	− 0.007	0.070	− 0.097	.923
Sex	3.846	0.430	8.951	< .001	3.921	0.427	9.178	< .001
SES-medium	− 0.438	0.756	− 0.579	.563	0.173	0.761	0.227	.820
SES-high	− 1.679	0.778	− 2.158	.031	− 0.904	0.789	− 1.146	.252
SES-highest	− 2.638	0.778	− 3.390	< .001	− 1.875	0.789	− 2.378	.018
Migration					2.179	0.441	4.947	.007*
*ΔR*^*2*^	*R*^*2*^ = .053, *F*(5, 1800) = 20.01, *p* < .001				*ΔR*^*2*^ = .013, *F*(1, 1799) = 24.468, *p* < .001			
*Self-concept*								
Intercept	63.598	1.136	55.988	< .001	63.452	1.176	53.960	< .001
Age	− 0.340	0.065	− 6.113	< .001	− 0.395	0.065	− 6.107	< .001
Sex	− 2.337	0.392	− 5.965	< .001	− 2.330	0.392	− 5.943	< .001
SES-medium	0.812	0.689	1.177	.239	0.866	0.699	1.239	.215
SES-high	1.751	0.709	2.469	.014	1.820	0.724	2.514	.012
SES-highest	2.695	0.710	3.797	< .001	2.762	0.724	3.817	< .001
Migration					0.195	0.404	0.481	**.630**^***ns***^
*ΔR*^*2*^	*R*^*2*^ = .046, *F*(5, 1800) = 17.27, *p* < .001				*ΔR*^*2*^ = .0001, *F*(1, 1799) = 0.2318, *p* = .630			

*B*, unstandardized regression coefficient*. SE*, standard error of *B*. Sex, 1 = female, 0 = male. SES, parental socio-economic status. Migration status: 1 = immigrant background, 0 = no immigrant background. Uncorrected *p*-values for estimates reflecting study hypotheses given in bold, alpha-error corrected thresholds applied to indicate significance at ***p > .001, **p > .01, *p > .05, *ns* = not significant

**Table 5:** Hierarchical regression analysis for self-concept predicting symptoms of depression and anxiety

Variable	Model 1				Model 2			
	*B*	*SE*	*t*	*p*	*B*	*SE*	*t*	*p*
*Depression*								
Intercept	27.732	1.236	22.440	< .001	58.053	1.840	31.553	< .001
Age	0.066	0.070	0.932	.352	− 0.123	0.064	− 1.923	.055
Sex	3.624	0.426	8.503	< .001	2.510	0.387	6.486	< .001
SES-medium	− 0.936	0.750	− 1.247	.213	− 0.549	0.675	− 0.813	.416
SES-high	− 1.983	0.772	− 2.569	.010	− 1.148	0.695	− 1.652	.099
SES-highest	− 2.432	0.772	− 3.149	.002	− 1.147	0.697	− 1.646	.100
Self-concept					− 0.477	0.023	− 20.677	< .001***
Δ*R*^2^	*R*^2^ = .046, *F*(5, 1800) = 17.27, *p* < .001				Δ*R*^2^ = .183, *F*(1, 1799) = 427.53, *p* < .001			
*Anxiety*								
Intercept	33.395	1.246	26.805	< .001	51.399	1.994	25.784	< .001
Age	− 0.010	0.071	− 0.139	.890	− 0.122	0.069	− 1.758	.079
Sex	3.846	0.430	8.951	< .001	3.185	0.419	7.594	< .001
SES-medium	− 0.438	0.756	− 0.579	.563	− 0.210	0.731	− 0.285	.776
SES-high	− 1.679	0.778	− 2.158	.031	− 1.183	0.753	− 1.571	.116
SES-highest	− 2.638	0.778	− 3.390	< .001	− 1.876	0.755	− 2.484	.013
Self-concept					− 0.283	0.025	− 11.332	< .001***
Δ*R*^2^	*R*^2^ = .053, *F*(5, 1800) = 20.01, *p* < .001				Δ*R*^2^ = .063, *F*(1, 1799) = 128.4, *p* < .001			

*B,* unstandardized regression coefficient*. SE,* standard error of *B*. Sex, 1 = female, 0 = male. *t*, *t*-value. *p*, *p*-values for *t*-tests on regression coefficient and intercept. *SES*, parental socio-economic status. *R*^*2*^ and *F*-tests for differences to intercept-only models. Uncorrected *p*-values for estimates reflecting study hypotheses in bold, alpha-error corrected thresholds applied to indicate significance at ***p > .001, **p > .01, *p > .05

### Moderated mediation analyses with the outcome depression (Hypotheses 3 and 4)

We predicted that age would moderate the indirect effect of the migration status on depression via self-concept (see Fig. 1a). The migration status did not influence the self-concept (path *a*). We consistently did not substantiate an overall indirect effect based on bootstrap confidence intervals (i.e., reduction of predictive value from path c to path c’). More specifically, we found no indirect effects of the focal predictor migration status on symptoms of depression for both age groups (children: *indirect effect* = − 0.016, 95% CI: − 0.358, 0.350; adolescents: *indirect effect* = − 0.020, 95% CI: − 0.485, 0.439). Self-concept thus did not represent a mediator for the demonstrated link between migration status and symptoms of depression. These findings contradicted the expectations of H3. However, the effect of self-concept on symptoms of depression depended on age as evidenced by the statistically significant interaction between *M* and *W* (path *b*_*3*_). While there was a negative link between self-concept and symptoms of depression in both age groups, this effect was stronger in the older group (children: *ß* = − 0.450, *p* < 0.001; adolescents: *ß* = − 0.587, *p* < 0.001). These findings were in line with H4 as a lower self-concept had a higher impact on increased symptom levels of depression during adolescence (i.e., participants older than 14 years of age). For further details see Table 6.

**Table 6:** Model coefficients for the moderated mediation models predicting symptoms of depression or anxiety

Antecedent	Consequent							
	*M* (Self-concept)				*Y* (Depression)			
	Path	Coeff	*SE*	*p*	Path	Coeff	*SE*	*p*
*X* (Migration status)	*a*	.035	.399	.931	*c’*	1.350	.384	< .001
*M* (Self-concept)	_	_	_	_	*b*_*1*_	-.450	.031	< .001
*W* (Age)	_	_	_	_	*b*_*2*_	4.767	1.794	.008
*M* × *W*	_	_	_	_	*b*_*3*_	-.138	.045	.003*
Constant	*i*_*M*_	38.569	.259	< .001	*i*_*Y*_	26.155	1.268	< *.*001
Model	*R*^*2*^ = -.000				*R*^*2*^ = .229			
	*F* (1, 1814) = .001, *p* = .931				*F* (4, 1811) = 134.439, *p* < .001			

Antecedent	Consequent							
	*M* (Self-concept)				*Y* (Anxiety)			
	Path	Coeff	*SE*	*p*	Path	Coeff	*SE*	*p*
*X* (Migration status)	*a*	.040	.398	.922	*c’*	2.287	.421	< .001
*M* (Self-concept)	_	_	_	_	*b*_*1*_	-.242	.034	< .001
*W* (Age)	_	_	_	_	*b*_*2*_	6.045	1.963	.002
*M* × *W*	_	_	_	_	*b*_*3*_	-.169	.050	< .001**
Constant	*i*_*M*_	38.551	.260	.000	*i*_*Y*_	22.679	1.389	< .001
Model	*R*^*2*^ = .000				*R*^*2*^ = .103			
	*F* (1, 1820) = .001, *p* = .922				*F* (4, 1817) = 52.091, *p* < .001			

Left column: Path *a* for predictor *X* (migration status) to mediator *M* (self-concept) calculated according to Hayes [56], *R*^*2*^ and *F*-tests for differences to intercept-only model. Right column: Full model paths (*c’*, *b*_*1*_, *b*_*2*_, *b*_*3*_) in moderated mediation models with symptom scores of depression or anxiety as consequent as illustrated in Fig. 1 Panel B), adjusted *R*^*2*^ and *F*-test for differences to intercept-only model. See Results section for estimates on conditional effects. *M*, mediator*. W,* moderator*. M* × *W,* interaction effect. *SE*, standard error*.* Uncorrected *p*-values for estimates reflecting study hypotheses in bold, alpha-error corrected thresholds applied to indicate significance at ***p > .001, **p > .01, *p > .05

### Moderated mediation analyses with the outcome anxiety (Hypotheses 3 and 4)

We predicted that age would moderate the indirect effect of migration status on symptoms of anxiety via self-concept (see Fig. 1a). The migration status did not affect the self-concept (path *a*). The indirect effect as estimated by bootstrap confidence intervals did not yield an effect. Specifically, we found no indirect influence of the focal predictor migration status on symptoms of anxiety for both age groups (children: *indirect effect* = − 0.009, 95% CI: − 0.198, 0.193; adolescents: *indirect effect* = − 0.411, 95% CI: − 0.345, 0.307). Self-concept thus did not function as a mediator for the demonstrated link between migration status and symptoms of anxiety. These findings similarly contradicted the expectations of H3. However, the effect of self-concept on symptoms of anxiety depended on age, as evidenced by the statistically significant interaction between *M* and *W* (path *b*_*3*_). While there was an overall negative link between self-concept and symptoms of anxiety, this link was consistently stronger in the older group (children: *ß* = − 0.242, *p* < 0.001; adolescents: *ß* = − 0.411, *p* < 0.001). These findings were in line with H4 as a lower self-concept had a higher impact on increased symptom levels of anxiety during adolescence. Overall, the moderated mediation model explained more variance of the outcome variable depression (R^2^ = 0.229) than of the outcome variable anxiety (R^2^ = 0.103). For further details see Table 6.

## Discussion

This study integrated perspectives of developmental science and clinical child psychology to examine the self-concept as an intra-individual correlate of mental health disparities between immigrant and non-immigrant youth throughout youth development. We could support that immigrant youth report more symptoms of depression and anxiety than non-immigrant youth and could also show that a lower self-concept is linked to such symptoms. However, our analyses did not yield self-concept as a significant mediator for these associations. Despite this null effect we found that age moderated the association between self-concept and internalizing mental health problems. We discuss study evidence along the study hypotheses.

### Immigrant children and adolescents experience more symptoms of depression and anxiety than non-immigrants

We hypothesized that having a migration background would link to increased levels of internalizing mental health problems. Indeed, migration status predicted symptoms of depression as well as anxiety. These findings contribute evidence to the ongoing debate whether migration status is linked to an increased risk for mental health problems (as hypothesized in this study), or alternatively facilitates processes of resilience. Contrary to our study propositions, some evidence on adolescent students in several European and other high-income countries substantiated the “immigrant (health) paradox”, that is, immigrant youth have a better mental health [59–62]. Based on a large dataset, our findings are however more consistent with our initial expectations of linking the migration status to lower mental health. Our evidence is supported by a stream of literature on the “mental health gap” between immigrant and non-immigrant youth [63, 64]. Possible explanations for the opposing propositions in the literature open two pathways. First, while many of the previous studies focus on immigrants from specific origin countries, our sample was more likely drawn from general immigrant populations in Germany including a variety of countries, ethno-cultural origins as well as immigrant generations. Through collecting data in non-exclusive classroom interviews, we did not limit our immigrant sample to specific groups. Our study sample thus more likely reflects the current and diverse population of children and adolescents with immigrant status in Germany. Some previous evidence suggests that the “immigrant health paradox” could encompass first-generation immigrants only [65]. Second, as our data was collected in school contexts, the identified mental health disparities might reflect consequences of social and ethnocultural discontinuities that immigrant children and adolescents can experience between home and school contexts [32].

### Self-concept does not mediate the link between migration status and depression/anxiety

We found that a lower self-concept was overall linked to increased symptom levels of depression and anxiety. This finding is in line with our expectations and previous studies on children and adolescents from general populations [28–30]. Based on the study sample, these findings moreover suggest that such associations could be generalizable to children and adolescents with diverse immigrant backgrounds.

We however did not find differences between immigrant and non-immigrant youth regarding their self-concepts. Correspondingly, our moderated mediation models did not substantiate a lower self-concept as a mediator for the link between migration status and internalizing mental health problems. These findings contradicted our expectations of a link between migration status and self-concept. At the same time, migration status significantly accounted for higher levels of internalizing mental health problems in our sample (H2). There are two approaches to explain such patterns of expected and unexpected findings. First, more specific dimensions of the self-concept, better reflecting a compartmentalized self-structure, rather than a global, unidimensional construct, merely reflecting an integrated self-structure, could better explain links between migration status and internalizing mental health problems. As a compartmentalized structure of the self was previously linked to lower mental health status and we substantiated fewer mental health problems for immigrant youth in our study, immigrant youth could have less accurately reported on their self-concepts using this global measure. Besides, the most relevant compartments of the self-concept that distinguish immigrant and non-immigrant youth may have not been in their cognitive foci when answering the self-concept measure. In a school context, compartments of the self-concept relating to academic components [66] likely influence cognitions when examining oneself. However, specific components of the self-concept, such as self-esteem or emotional stability, could have been relatively less salient for immigrant children and adolescents during study participation—even though such domains might be more strongly linked to internalizing mental health problems. Second, the self-concept, either in global or domain-specific operationalization, is not directly associated with the migration status as we hypothesized. Considering the deductions from the coexisting immigrant paradox, immigrant youth could be better off in some health-associated psychological domains (including the self-concept), while they simultaneously show increased symptom levels in others (including symptoms of depression and anxiety).

### Age moderates the link between self-concept and depression/anxiety

Consistent with our expectations, we found that adolescents’ internalizing mental health problems are more strongly affected by a lower self-concept in our sample. The influence of the self-concept on the participants’ mental health status was moderated by age, meaning a lower self-concept seemed to impact adolescents more strongly than children. Considering age as a proxy for youth development, our evidence highlights the importance of developmental processes for understanding the relationship between self-concept and mental health. Consistent to this model and to our evidence, a previous study found older age and more internalizing mental health problems to predict lower self-concept in a multi-ethnic youth group in the United States [67].

Moreover, behavioral and cognitive covariations of the self-concept with children’s and adolescents’ age need additional consideration when interpreting our findings. For young children, the self-concept is less validated by outside cues and tends to be more positive in general [68]. Marsh, Parada, Yeung and Healey [69] postulated that younger children defend their self-concept with “troublemaking behavior” (i.e., socially directed strategies). While our study focus was on symptoms of depression and anxiety, the specific symptoms of mental health problems with either externalizing or internalizing nuances that are associated with a low self-concept could thus vary as a function of age. Another approach to explaining age differences in the self-concept focuses on its intrapersonal organization. In a study with youth suffering from attention deficit hyperactivity disorder and internalizing mental problems [67], adolescents reported a lower self-concept than children. Study authors discussed whether such differences were either due to developmental processes influencing the organization of the self-concept or due to the stability of problem behavior which could have negatively affected the self-concept over time. Also, in our study, continuous social withdrawal could thus have limited the reception of environmental cues that are required for establishing a stable and positive self-concept during adolescence.

### Strengths, limitations and future research

There were several limitations that underline the need for future research on the identification of within-person mediators for the mental health disparities between immigrant and non-immigrant youth. First of all, the sample was collected via self-report measures in school settings. To further validate our findings and substantiate generalization, we need replication studies with more sophisticated designs, such as using multi-informant approaches and longitudinal data collections from middle childhood to late adolescence. Second, the self-concept was measured via self-report questionnaires using a unidimensional construct applicable to a variety of social settings. The use of multi-dimensional measures, for example the Self-Description Questionnaire II [45], could better assess compartmentalized structures of the self-concept. Third, the BYI-II subscales were administered to a highly heterogenous group with immigrant backgrounds. To date, constructs as operationalized in these inventories still have to prove psychometric fidelity and measurement invariance, especially with regard to respondents’ ethnocultural backgrounds. Nonetheless, the present study has several strengths. It combined clinical child psychology and developmental science perspectives as it investigated the impact of the self-concept on symptoms of depression and anxiety from middle childhood to late adolescence. We included immigrant youth of the second- and third-generations from multiple origin countries to acknowledge youth’s diversity in current German society, while past research on adolescent mental health has extensively focused on first-generation immigrant populations [7, 20]. Regarding the study population, our evidence is based on a regionally and socio-economically controlled sample across the largest federal state in Germany and data was collected in a variety of different school types. However, we cannot fully preclude bias due to undetected mechanisms linked to socio-economic disparities. The parental socio-economic status of immigrant youth in our sample was on average lower in the immigrant sample. The parental socio-economic status was moreover linked to symptoms of anxiety which is consistent to previous findings.

Subsequent research needs to further investigate which within-person processes during childhood and adolescence mediate the link between migration status and internalizing mental health problems, possibly with regard to specific contexts of measurement. Beyond the self-concept, future studies could consider the children’s or their families’ cultural orientations including the significance of religion, or perceived discrimination at school. For example, Schunck, Reiss and Razum [70] provided evidence that perceived discrimination could mediate mental health disparities between immigrant and non-immigrant adults in Germany.

### Implications

Our study findings overall underlined the importance of the self-concept for the mental health of children and adolescents. Our findings moreover emphasized that a positive self-concept is especially relevant during adolescence [71]. Although our study did not directly address the practical relevance of these findings, previous intervention studies repeatedly demonstrated that a strong self-concept has positive implications for youth’s mental health in school contexts. Rousseau et al. [72] evaluated a school-based prevention program that uses arts and self-expression to strengthen the self-concept of immigrant and multi-ethnic youth. Children enrolled in this program reported higher levels of well-being in comparison to a control group. Consistent evidence for addressing students at a younger age was provided by De Bettignies and Goldstein [73]: Improvisational theatre classes in elementary schools were found to improve children’s self-concept and mitigate internalizing mental health problems.

## Conclusions

The proportion of immigrant children and adolescents in Germany has been increasing over recent years. Interdisciplinary practice and research integrating developmental science and clinical child psychology is required to not only consider highly diverse backgrounds among children and adolescents but also needs to acknowledge the fact that those from immigrant backgrounds have higher symptom levels of depression and anxiety. While our study supported such mental health disparities between immigrant and non-immigrant groups when assessed in school settings, data did not support that lower levels of the self-concept mediate these associations. Beyond replication studies to substantiate our findings, the search for underlying mediators needs to be continued with more specific research designs and additional focus on other migration-related candidate variables.

## Data Availability

The dataset supporting the conclusions of this article is available from the corresponding author and GS on reasonable request. Measures used for the primary outcomes can be obtained from the “Pearson Assessment & Information GmbH”.

## Abbreviations

BAI-Y: Beck Anxiety Inventory for Youth
BDI-Y: Beck Depression Inventory for Youth
BSCI-Y: Beck Self-Concept Inventory for Youth
BYI-II: Beck Youth Inventory Second Edition
CIs: Confidence intervals
ISCED: International Standard Classification of Education
SES: Socio-economic status
